# Enzymatic Synthesis of Ascorbyl Palmitate in a Rotating Bed Reactor

**DOI:** 10.3390/molecules28020644

**Published:** 2023-01-08

**Authors:** Jessica Holtheuer, Luigi Tavernini, Claudia Bernal, Oscar Romero, Carminna Ottone, Lorena Wilson

**Affiliations:** 1 School of Biochemical Engineering, Pontificia Universidad Católica de Valparaíso, Avenida Brasil 2085, Valparaíso 2340000, Chile; 2 Laboratorio de Tecnologia Enzimatica para Bioprocesos, Instituto de Investigación Multidisciplinario en Ciencia y Tecnología, Universidad de La Serena, Raúl Bitran 1305, La Serena 1700000, Chile; 3 Bioprocess Engineering and Applied Biocatalysis Group, Department of Chemical Biological and Environmental Engineering, Universitat Autònoma de Barcelona, 08193 Bellaterra, Spain

**Keywords:** rotating bed reactor, lipase, enzymatic synthesis, ascorbyl palmitate

## Abstract

Ascorbyl palmitate, an ascorbic acid ester, is an important amphipathic antioxidant that has several applications in foods, pharmaceuticals, and cosmetics. The enzymatic synthesis of ascorbyl palmitate is very attractive, but few efforts have been made to address its process scale-up and implementation. This study aimed at evaluating the enzymatic synthesis of ascorbyl palmitate in a rotating basket reactor operated in sequential batches. Different commercial immobilized lipases were tested, and the most suitable reaction conditions were established. Among those lipases studied were Amano Lipase PS, Lipozyme^®^ TL IM, Lipozyme^®^ Novo 40086, Lipozyme^®^ RM IM and Lipozyme^®^ 435. Initially, the enzymes were screened based on previously defined synthesis conditions, showing clear differences in behavior. Lipozyme^®^ 435 proved to be the best catalyst, reaching the highest values of initial reaction rate and yield. Therefore, it was selected for the following studies. Among the solvents assayed, 2-methyl-2-butanol and acetone showed the highest yields, but the operational stability of the catalyst was better in 2-methyl-2-butanol. The tests in a basket reactor showed great potential for large-scale application. Yields remained over 80% after four sequential batches, and the basket allowed for easy catalyst recycling. The results obtained in basket reactor are certainly a contribution to the enzymatic synthesis of ascorbyl palmitate as a competitive alternative to chemical synthesis. This may inspire future cost-effectiveness studies of the process to assess its potential as a viable alternative to be implemented.

## 1. Introduction

Ascorbyl palmitate (AsPa) is an antioxidant derived from ascorbic acid (AA) that represents a feasible substitute which can be used in fatty matrices with applications in the food, pharmaceutical and cosmetic industries, providing the same essential properties as ascorbic acid [1,2,3,4,5]. The production of ascorbyl palmitate is accomplished mainly by chemical synthesis. Nevertheless, by-product formation may cause serious environmental risk (also complicating purification), which in turn compromises product quality and reduces process efficiency. Enzymatic synthesis, on the contrary, prevents by-product formation thus facilitating purification and reducing negative environmental impact, being a sustainable approach with great potential as a process alternative to chemical synthesis at industrial level [3]. Although enzymatic synthesis is very attractive, only a handful of studies address reaction conditions in reactor aiming at process scale-up and implementation [6].

The enzymatic route to ascorbyl palmitate synthesis is catalysed by a lipase. Lipases are quite versatile catalysts with numerous advantages that have secured their application in a broad range of reactions of commercial interest [7,8]. These enzymes have been immobilized on a wide variety of supports using different strategies, allowing their stabilization and reuse under operating conditions. The numerous published and patented works on this topic provide testimony of the great potential of these catalysts in applications such as esterification and hydrolysis of oils, with many interesting results that make use of ultrasound, supercritical fluids, and ionic liquids as novel strategies to maximize reaction performance [7].

Not many studies in the literature treat the behaviour of immobilized catalysts in reactors or the factors relevant for process scale-up. Among the latter, it is necessary to consider the mode of operation (batch, fed batch or continuous), characteristics of the substrate, characteristics of the biocatalyst such as resistance and the need for organic solvents. The most used reactors in processes involving immobilized lipases are batch stirred tanks (STR), followed by packed bed reactors (PBR) operated in continuous mode. PBRs stand out for their scale-up ease and low shear stress that prevents enzymatic desorption [9].

In the case of immobilized enzymes, it is very important to assess the mechanical stability of the support. For example, agitation in a STR could damage the support due to shear stress, which may lead to enzyme leakage and activity loss [10]. The search for good strategies to protect the enzymes and facilitate catalyst recovery in STRs have been advanced using basket reactors with very good results [11,12]. In addition, Hajar et al. [13] evaluated the effects of mass transfer, a key factor in heterogeneous catalysis, on the synthesis of an n-butyl oleate ester using Novozym 435 in a laboratory scale basket reactor (SBR). They carried out a statistical study for the determination of parameters considering a bi-bi ping-pong reaction mechanism, yielding values for the Thiele modulus and the Damköhler number that showed the absence of internal and external diffusion limitations over the reaction.

The aim of this work was to study the enzymatic synthesis of ascorbyl palmitate in a basket reactor operated in consecutive batches in order to evaluate the potential of this enzymatic system for large-scale operation. To this end, we evaluated different commercial immobilized lipases and tested different conditions to maximize reaction performance. The lipases studied were Amano Lipase PS, Lipozyme^®^ TL IM, Lipozyme^®^ Novo 40086, Lipozyme^®^ RM IM and Lipozyme^®^ 435. Once the appropriate enzyme was selected, the best reaction conditions were defined, allowing thereafter for the study of the behaviour of the biocatalyst in a sequential batch operation.

## 2. Results and Discussion

### 2.1. Biocatalyst Screening

A study of five immobilized commercial lipases was conducted by measuring hydrolysis activity of the artificial substrate pNPB and the synthesis activity of AsPa under the conditions described in Section 3.2 and Section 3.3. The results obtained are shown in Table 1.

From Table 1, it is possible to see that Amano Lipase PS and Lipozyme^®^ TL IM show the highest values of hydrolysis activity. However, the highest AsPa synthesis activity is obtained with Lipozyme^®^ 435. Interestingly, the other enzymes exhibit very similar values of synthesis activity.

Lipozyme^®^ 435 is the Candida antarctica lipase B enzyme immobilized on Lewatit VP OC 1600 resins via interfacial activation. It is considered an ideal enzyme catalyst in terms of its recycling capabilities [14]. This catalyst has shown great stability in organic solvents and has been applied in a broad range of reactions [4,15,16,17].

### 2.2. Comparison of Different Biocatalysts on Ascorbyl Palmitate Synthesis

The above five commercial immobilized lipases were then tested on AsPa synthesis. Previously, sieve concentration and AA adding strategy were studied using the conditions proposed by Tufiño et al. [3] as a starting point. Due to the formation of water during the reaction and its negative effect on the performance of lipases, different concentrations of molecular sieve were evaluated. Preliminary results showed that adding 180 mg of activated molecular sieve (3.5%) at the beginning of the reaction was the most adequate strategy to reach maximum yield (see Section 3.7). As a comparison, in the work of Costa et al. [18], reactions for the enzymatic synthesis of ascorbyl oleate were carried out using 20% molecular sieve.

Regarding the strategy of AA addition, it was possible to witness that adding the entire concentration of substrate at the beginning caused the medium to turn dark, a sign of dehydroascorbic acid presence due to AA oxidation [18,19]. Consequently, adding AA in two steps was evaluated, favoring reaction conversion and preventing AA oxidation. Fed-batch operations have been reported in several studies as a solution for cases in which substrates cause enzyme inhibition. A clear example is the synthesis of biodiesel where methanol exerts inhibition [20]. Another case is the synthesis of benzyl butyrate by direct esterification using Novozym^®^ 435. In the latter, the feeding strategy of propionic acid improved the performance of immobilized lipase, facilitating ester conversion and allowing also recycling the biocatalyst throughout many esterification cycles [21].

**Figure 1:**
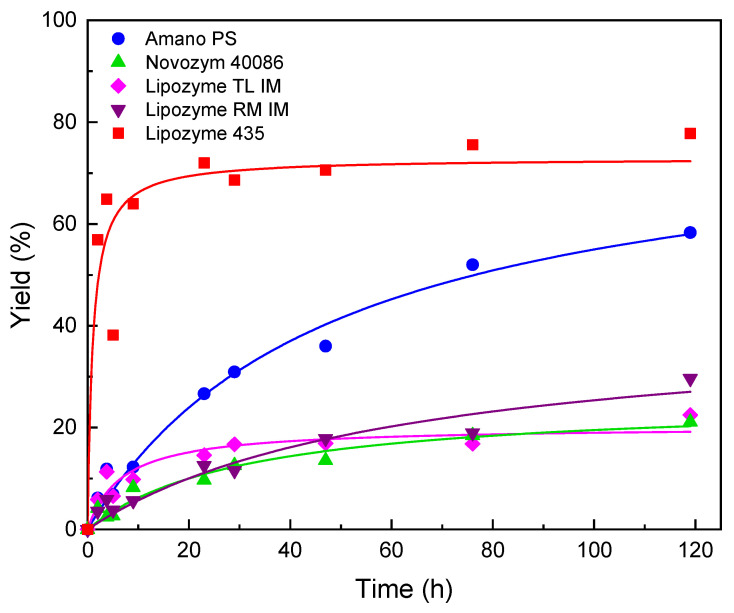
Synthesis of ascorbyl palmitate with commercial immobilized lipases. The reaction was carried out at ascorbic acid to palmitic acid ratio 1:8 at 55 °C with 180 mg molecular sieve in 5 mL 2-methyl-2-butanol. Ascorbic acid was added at times 0 h (30 mg) and 4 h (30 mg).

Once the amount of sieve and the adding strategy of AA were established, AsPa synthesis was carried out using the five commercial immobilized lipases. Figure 1 shows AsPa production kinetics with the different enzymes proposed under the previously defined conditions.

As shown in Figure 1, after 120 h of reaction the best results were obtained with Lipozyme 435 (77.7% yield) and Amano PS (58.3% yield). The other immobilized catalysts showed a poor performance reaching less than 30% of final yield. Additionally, initial synthesis reaction rates are presented in Table 2. As expected, Lipozyme 435 showed significantly higher activity than the other immobilized catalysts (about 18–8 fold). This correlates well with the initial assessment of synthesis activity (Table 1), showing the predictive value of this simple and fast catalyst screening approach.

Results of the AsPa synthesis are consistent with studies of *Candida antarctica* lipase in similar reactions and conditions [18]. For example, the commercial lipase NS 88011, a *Candida antarctica* lipase immobilized on a hydrophobic polymer resin, was used for the synthesis of ascorbyl oleate, reaching a maximum conversion of 50% at optimized conditions of 70 °C, 30% enzyme loading, and ascorbic acid to oleic acid molar ratio 1:9 [18]. These represent much more drastic conditions than those assayed in this work risking enzyme inactivation.

**Figure 2:**
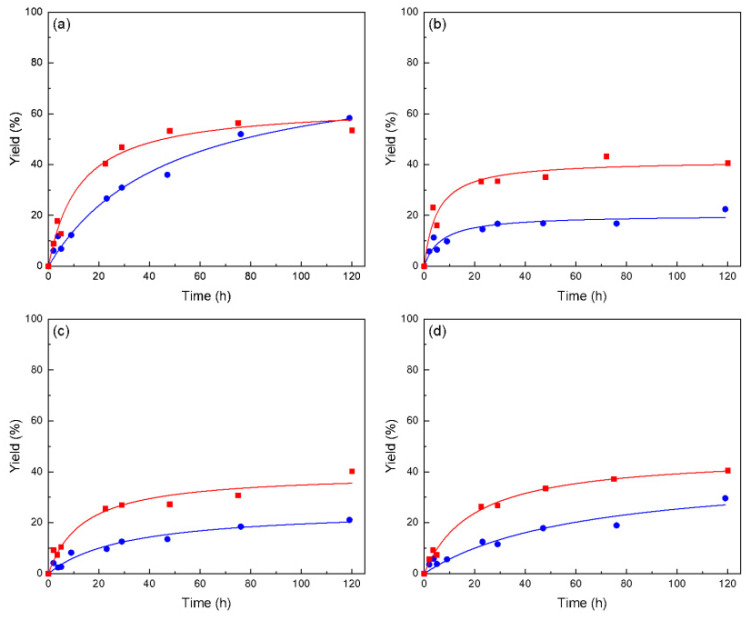
Synthesis of ascorbyl palmitate at biocatalyst concentrations of 60 mg (●) and 180 mg (■) using commercial lipases: Amano PS (**a**), Lipozyme TL IM (**b**), Novozym 40086 (**c**), Lipozyme RM IM (**d**). Ascorbic acid to palmitic acid ratio 1:8, 55 °C, 180 mg molecular sieve in 100% 2-methy-2-butanol. Ascorbic acid was added at times 0 h and 4 h of reaction (30 mg each time).

Considering the low initial reaction rate of four of the five catalysts assayed (Amano PS, Lipozyme TL IM, Novo 40086 and RM IM), we studied the synthesis of ascorbyl palmitate under the same reaction conditions but increasing the amount of these biocatalysts by threefold, expecting to witness an improvement on conversion. The results are presented in Figure 2.

As can be seen in Figure 2, even though yields improved in almost all cases, they did not increase as expected. This is consistent with the initial reaction rates shown in Table 2. These lipases display good hydrolysis behavior, but the AsPa synthesis conditions seem to be detrimental for their activity (see Table 1). Based on these results and having in mind the large-scale production of the antioxidant, Lipozyme^®^ 435 lipase was selected for the following study. Tufiño et al. [3] also reported *Candida antarctica* lipase as a preferable biocatalyst for AsPa synthesis as compared to the lipase of *P. stutzeri* immobilized in silica.

It is not always the case that *Candida antarctica* lipase yields the best results as compared to other enzymes. For instance, in a study reported by Zhu et al. [22], they compared Novozym^®^ 435 and Lipozyme TL-IM on the interesterification of a palm stearin and vegetable oil blend in order to enhance its physicochemical characteristics, with Lipozyme TL-IM delivering the best results. On another work, Lipozyme RM-IM showed best performance in the synthesis of capric acids via batch acidolysis in solvent-free medium [23].

### 2.3. The Effect of Solvent and Temperature on Ascorbyl Palmitate Synthesis by Lipozyme 435

**Figure 3:**
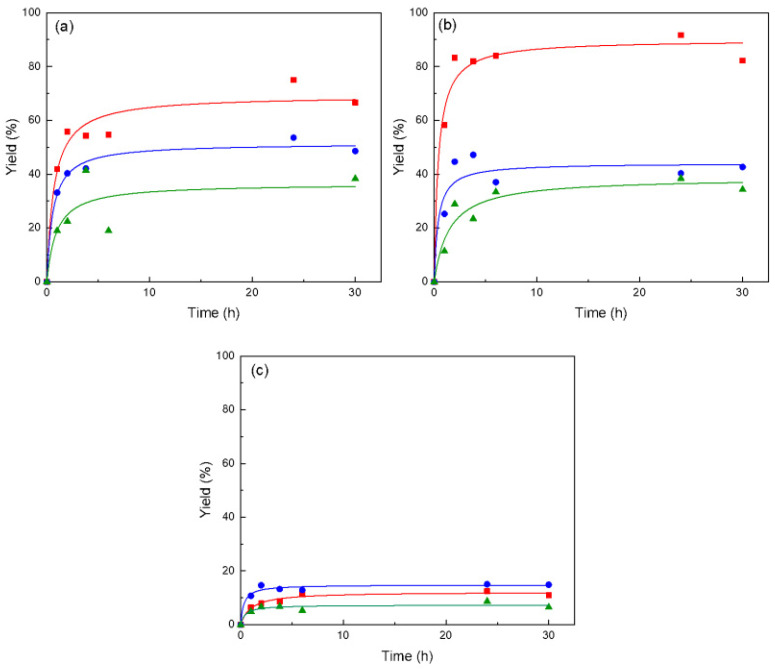
Effect of solvent and temperature on the synthesis of ascorbyl palmitate catalyzed by Lipozyme 435. Solvents: 2-methy-2-butanol (**a**), acetone (**b**) and ter-butanol (**c**). Temperatures: 45 °C (▲), 50 °C (●) and 55 °C (■). Reactions were carried out in 5 mL solvent media at substrate ratio 1:8, with 60 mg ascorbic acid added in two steps (time 0 h and after 4 h). Other reactants added were 698 mg palmitic acid, 60 mg Lipozyme 435, and 180 mg molecular sieve.

Further studies of the reaction conditions were carried out assessing the effect of solvent and temperature. Three different solvents were tested, 2-methyl-2-butanol (2M2B), acetone and ter-butanol. The temperature of synthesis was evaluated between 45 and 55 °C. One important criterion for the selection of a solvent is how easy it can be recovered and reused. In this case, recovery of all of the solvents tested was feasible and easy. Figure 3 shows the yields obtained in the conditions evaluated.

As can be seen in Figure 3, the highest yield was achieved in acetone at 55 °C obtaining over 80% (22.6 g/L). Acetone has shown to be a good solvent for esterification, in particular using immobilized *Candida antartica* lipase, as in the synthesis of ascorbyl acetate [24] and alkyl esters of prunin [25].

High yields were obtained also in 2M2B, accentuating the effect of temperature, as was also the case of acetone above, meaning that yield increases as temperature rises. These results confirm what we showed in a previous work, where 2M2B resulted as an excellent candidate for ascorbyl palmitate esterification using commercial or made-in-house immobilized lipases [3].

Ter-butanol was dismissed due to the low conversion achieved. Results in ter-butanol were unexpected. In contrast to this work, Balen et al. [26] obtained conversions of linoleic acid into AsPa of 90% in ter-butanol using Novozym^®^ 435. In another example, Nehdi et al. [27] achieved yields as high as 97.8% in the production of biodiesel with Novozym^®^ 435 in ter-butanol medium. Also, Yadav et al. [17] reported 50% conversion of ascorbyl palmitate with Novozym^®^ 435 in ter-butanol. Furthermore, stability of this biocatalyst has also been reported to be favorable in ter-butanol, something that is key for the economic viability of the process. In studies with Novozym^®^ 435 for the synthesis of ascorbyl oleate, enzyme activity dropped to 50% only after 14 cycles in ter-butanol. The authors associated this loss to the effect of the solvent on the hydration layer of the enzyme [18].

As already mentioned, temperature exerts a significant effect in both 2M2B and acetone media, with the highest yield reached at temperature 55 °C. These conditions were selected to deepening studies on the stability of the catalyst.

### 2.4. Solvent Effect on the Operational Stability of the Biocatalyst

**Figure 4:**
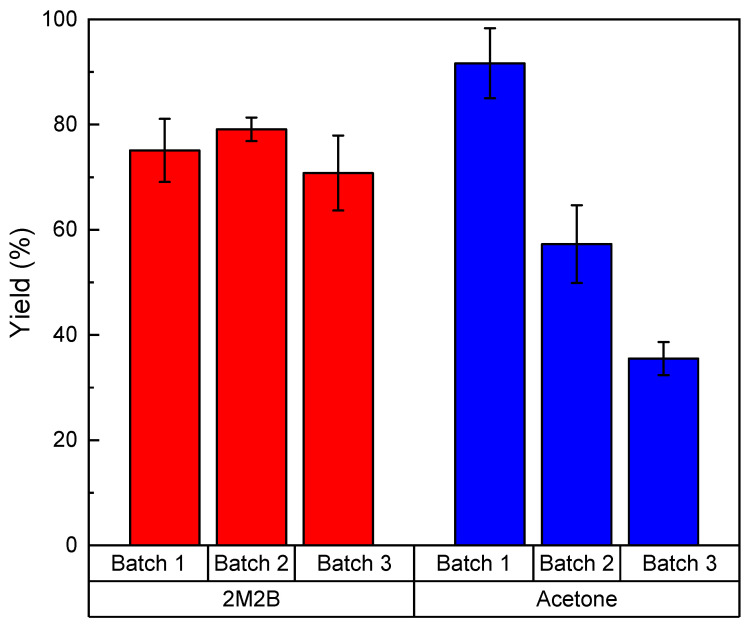
Operational stability of Lipozyme^®^ 435 in the synthesis of ascorbyl palmitate at 55 °C in 2-methyl-2-butanol and acetone media. Results are depicted as mean ± standard deviation.

In order to study the effect of the type of solvent on the biocatalyst stability, an operational stability test was carried out consisting on three sequential batches of antioxidant synthesis in 2M2B and acetone at 55 °C. The results are shown in Figure 4.

Each batch was conducted for 72 h, a period much longer than the time needed to reach the maximum yield. Therefore, more batches could have been carried out during the same time. Figure 4 shows that although a higher yield is achieved with acetone in the first batch, stability decreased substantially, and after the third batch using the enzyme is no longer feasible (30% yield approximately). Conversely, the reaction yield remained at about 70% after three batches in 2M2B, showing a much higher stability. Consequently, 2M2B was selected as the most adequate solvent for the next assays in rotating bed reactor.

### 2.5. Synthesis of Ascorbyl Palmitate in a Rotating Bed Reactor in a Sequential Batch Operation

After establishing the biocatalyst and the reaction conditions, the synthesis of the antioxidant was carried out in a rotating bed reactor. Rotation provides optimal mass transfer during the reaction, which translates into higher yields. The reactor basket allows for catalyst recycling and prevents catalyst breakup caused by stirring. The basket has a mesh cut of 100 µm, suitable for the retention of Lipozyme^®^ 435, which has an average particle size of 315–1000 µm [14].

Each batch duration was set at 30 h, using the biocatalyst 4 times under the conditions indicated in Section 3.9. No activity or yield loss was detected in the evaluated time. It is noteworthy that the yields achieved are the highest among those obtained in this study and those reported in the literature. This could be the outcome of the mixing provided by the reactor agitator, which enhances mass transfer as mentioned.

**Figure 5:**
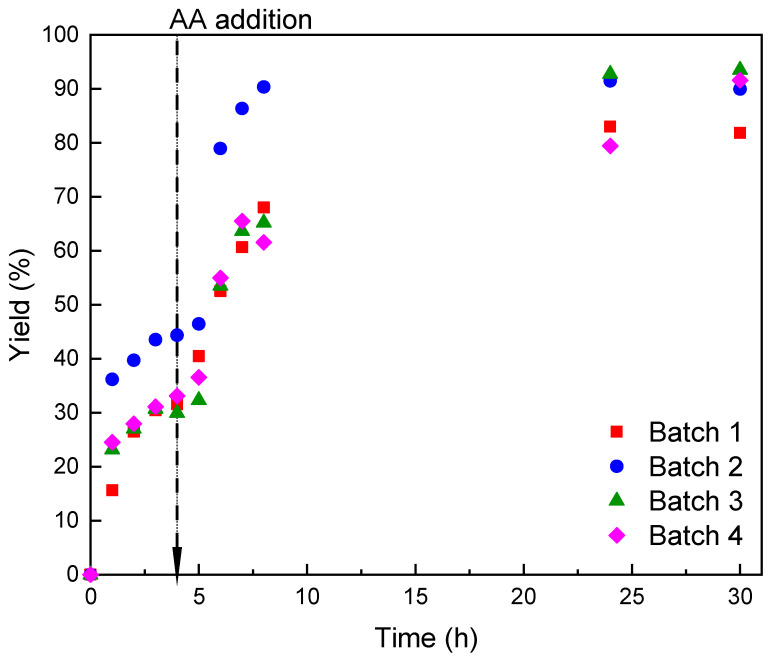
Synthesis of ascorbyl palmitate in a rotating bed reactor operated in sequential batches using lipase Lipozyme^®^ 435. Reaction conditions: substrates ratio 1:8, 55 °C, 290 rpm, 1.8 g molecular sieve in 2-methyl-2-butanol.

Overall, the second batch achieved higher yields than the first batch at comparable times, which could be attributed to the interfacial activation suffered by lipases. Rodrigues et al. [28] argue that the seeming activation of Novozym^®^ 435 lipase is the result of catalyst rupture due to mechanical agitation. This may be occurring to a lesser extent in the basket reactor, explaining the performance boost witnessed in the second batch. Although the reaction was tracked for 30 h, it can be seen from Figure 5 that full yield is reached by 15 h approximately, marking the operational end of each batch.

As can be seen in Table 3, yields of ascorbyl palmitate synthesis remained high during the four batches. Space time yield (STY) showed excellent values in each batch averaging 0.84 (g_AsPa_ g^−1^ L^−1^). This represents 1.8 to 4.2 fold increase as compared to previous studies on AsPa synthesis [3,29]. Also remarkable was the biocatalyst yield, obtaining 8.4 (g_AsPA_ g_catalyst_^−1^). This value can be substantially improved by recycling the catalyst into more batches considering that no significant loss of catalyst activity is observed. To our knowledge, this is the first study of biocatalyst reuse for the synthesis of AsPa.

The results achieved in this work represent a contribution to enzymatic synthesis studies mediated by lipases. Higher yields and greater productivity were accomplished than those achieved in previous works [3,29,30].

## 3. Materials and Methods

### 3.1. Materials

p-nitrophenol (pNP), p-nitrophenyl butyrate (pNPB), ascorbyl palmitate and the 3Å molecular sieve were purchased from Sigma-Aldrich (St. Louis, MO, USA). Palmitic acid (PA) was acquired from Loba Chemie (Mumbai, India). Acetone, acetonitrile, hexane, ethyl acetate, 2-methyl-2-butanol, monopotassium phosphate and ascorbic acid were of the highest available purity and were purchased from Merck (Darmstadt, Germany).

The biocatalysts used in this research are shown in Table 4, all generously donated by Novozyme Spain.

### 3.2. Hydrolysis Activity Assay

The hydrolytic activity of the commercial lipases was determined by measuring pNP resulting from the hydrolysis of pNPB. Briefly, 19.8 mL of a phosphate buffer solution (25 mM pH 7.0) containing 5 mg/mL of enzyme was incubated in a bath at 30 °C. The reaction was started by adding 0.2 mL of pNPB (50 mM in acetonitrile). Samples of 1 mL were taken and filtered every 30 s during 3 min and then subjected to absorbance measurements at 348 nm in spectrophotometer. Activity was determined considering a molar extinction coefficient ε = 5.15 mM^−1^ cm^−1^. One lipase hydrolytic activity unit (IU_H_) was defined as the amount of enzyme producing 1 µmol of pNP per minute at pH 7.0 and 30 °C. Assays were conducted in triplicates.

### 3.3. Synthesis Activity Assay

The initial synthesis reaction rate was measured by keeping track of the reaction kinetics for 24 h. Samples were taken and filtered every 30 min for the first 3 h and then at 5 and 24 h of reaction. The assay was conducted at 45 °C 150 rpm in 5 mL 2M2B at substrate molar ratio 1:5 (60 mg AA, 436 mg PA) adding 60 mg of biocatalyst and 70 mg of activated molecular sieve. Before the reaction, the substrate solution was prepared by solubilizing AA in 2M2B for 1 h in shaker at 45 °C 150 rpm. The samples were analysed in HPLC measuring AsPA and AA. Activity was determined considering only those data below 20% conversion (first 4 h of reaction approximately). One synthesis activity unit (IU_S_) is defined as the amount of enzyme producing 1 µmol of AsPa per minute at pH 7.0 and 45 °C. Assays were conducted in triplicates.

### 3.4. Molecular Sieve Activation and Solvent Drying

Molecular sieves of 3Å were used for the drying of solvents and the synthesis of AsPa. The sieves were activated by vacuum drying in a centrifugal concentrator (SpeedVac SPD111 VP2, Thermo Scientific, Waltham, MA, USA) for 2 h at 35 °C and 1 h in vacuum.

The solvents used for the synthesis of AsPa were dried by contacting them with 3Å molecular sieves at 10% weight per solvent volume for 48 h.

### 3.5. HPLC Analysis of Reagents and Product

The quantification of AsPa and substrates AA and PA was carried out by HPLC with a C-18 column Kromasil C18, 5 μm, 4.6 mm × 150 mm (Analisis Vinicos S.L., Ciudad Real, Spain) and a UV–vis spectrophotometer (JASCO model AS-2089). Analyte separation was accomplished by using an acetonitrile:water mobile phase at 1 mL/min following a gradient schedule where the mobile phase was 60:40 *v*/*v* for the first 6 min and 95:5 *v*/*v* for the next 22 min. AA and AsPa retention times were 1.2 and 13.5 min respectively. Concentrations of AsPA, PA and AA were determined using standard curves previously elaborated in the concentration range from 0 to 4 mM.

### 3.6. Synthesis of Ascorbyl Palmitate with Different Commercial Immobilized Lipases

The synthesis of AsPa was conducted in shaker at 55 °C and 160 rpm based on the conditions reported by Tufiño et al. [3]. The reaction mixture contained 5 mL 2M2B, 180 mg of activated molecular sieve, 698 mg PA, and 60 mg AA added in two steps (30 mg initially and 30 mg after 4 h of reaction). The reaction started once 60 mg of commercial biocatalyst were added to the medium. Samples were taken during 120 h and subjected to HPLC analysis. Assays were conducted in triplicates. The yield (Y, %) was defined based on the content of AsPa as follows:

(1) $$Y\%=\frac{{\mathrm{AsPa}}_{S}}{{\mathrm{AsPa}}_{t}}\times 100$$

where,

AsPas: Concentration of synthesized ascorbyl palmitate, mM

AsPat: Theoretical concentration of ascorbyl palmitate at full ascorbic acid conversion, mM

The initial synthesis reaction rate corresponded to the slope of the initial AsPa concentration vs time readings (first 4 h of reaction approximately).

### 3.7. Evaluation of Reactions Conditions

This stage was carried out only with Lipozyme^®^ 435, which expressed the highest conversion yield at the reaction conditions reported by Tufiño et al. [3]. Three solvents were tested: 2M2B, acetone, and tert-Butyl alcohol, all dried using activated molecular sieve. In addition, temperatures 45 °C, 50 °C and 55 °C were assayed. The reaction mixture and other conditions were as described above (Section 3.6).

### 3.8. Operational Stability of Lipozyme 435

The operational stability assay of Lipozyme 435 was carried out in 5 mL solvent and 60 mg of enzyme per batch using alternatively 2M2B and acetone at 55 °C. Other conditions were those reported by Tufiño et al. [3]. Samples were taken for 73 h and analysed in HPLC. After each batch, the biocatalyst was filtered, recovered, and rinsed three times with the solvent used in the reaction. When the biocatalyst was not reused immediately, it was stored at 4 °C. Assays were conducted in triplicates.

### 3.9. Synthesis of Ascorbyl Palmitate in a Basket Reactor Operated in Sequential Batches

A 250 mL rotating bed reactor (RBR S2, Spinchem, Umeå, Sweden) was used [33]. Rotation in this reactor favours mass transfer and the basket allows for biocatalyst recovery. The synthesis was conducted at 290 rpm 55 °C in 150 mL 2M2B solvent with 20.9 g PA, 5.4 g molecular sieve, and 1.8 g AA added in two steps (900 mg at initial time and 900 mg after 4 h of reaction). The reaction started once 1.8 g Lipozyme^®^ 435 were added. AsPa concentration in the medium was traced for 30 h. Once each batch ended, the reaction media was removed and the solvent and product were recovered using a rotary evaporator (Rotavapor R-100, Buchi). The biocatalyst was rinsed with 2M2B without removing it from the basket. Finally, four batches were carried out in total under the aforementioned conditions.

## 4. Conclusions

The results in this study reveal the prime importance of adequate screening of the catalyst and the operating conditions for the synthesis of ascorbyl palmitate. Among the commercial immobilized catalysts assessed, Lipozyme^®^ 435 was the enzyme reaching the highest initial reaction rate, yield and productivity, securing its selection for the assays to follow. The study of the operating conditions showed that the solvent, sieve, and substrate addition in two steps, were the key process intensification factors for the success of the synthesis tests in reactor. The reactor basket allowed for enzyme recycling, and four repeated batches were performed obtaining over 80% yield in each batch. Such satisfactory results are arguably the outcome of the ideal mixing conditions provided by rotation that reduce mass transport limitations both inside and out of the basket containing the biocatalyst particles. The results obtained in this study in terms of reaction yield and catalyst recycling are an important contribution to lipase-catalyzed synthesis processes. Future studies will be necessary to elucidate how profitable and viable this technology can be for industrial application, considering not only catalyst cost, but also solvent recovery and product purification. The latter may involve considerable savings in purification equipment, operation costs (reactants, time, energy, labor, etc.), and stream treatment to comply with environmental regulations.

## Figures and Tables

**Table 1:** Hydrolysis and synthesis activity of commercial immobilized lipases.

Commercial Name	Hydrolysis Activity (IU_H_/g) *	Synthesis Activity (IU_S_/g) *
Amano Lipase PS	1063 ± 144	3.5 ± 0.7
Lipozyme^®^ TL IM	768 ± 106	2.5 ± 0.7
Lipozyme^®^ Novo 40086	93 ± 17	2.3 ± 0.4
Lipozyme^®^ RM IM	74 ± 10	2.6 ± 0.1
Lipozyme^®^ 435	78 ± 4.4	33.5 ± 3.5

* Hydrolysis (IU_H_) and synthesis activity (IUs) are defined in Section 3.2 and Section 3.3, respectively.

**Table 2:** Initial synthesis reaction rate of ascorbyl palmitate of the commercial immobilized lipases evaluated.

Commercial Name	Initial Reaction Rate μmol min^−1^ g^−1^	Final Yield (%)
Amano Lipase PS	90.8 ± 1.5	58.3
Lipozyme^®^ TL IM	84.5 ± 1.7	22.5
Lipozyme^®^ Novo 40086	72.6 ± 15.6	5.6
Lipozyme^®^ RM IM	41.4 ± 4.8	29.6
Lipozyme^®^ 435	770.6 ± 51.9	77.7

**Table 3:** Summary of the main metrics obtained in the synthesis of ascorbyl palmitate in a rotating bed reactor operated in sequential batches.

Metrics	Batch 1	Batch 2	Batch 3	Batch 4	Accumulated
Yield (%)	81.9	90.0	93.5	91.6	-
AsPa final concentration (g/L)	23.1	25.4	26.4	25.9	-
AsPa produced (g)	3.5	3.8	4.0	3.9	15.1
STY (g L^−1^ h^−1^)	0.77	0.85	0.88	0.86	0.84
Biocatalyst Yield (g g^−1^)	1.93	2.12	2.20	2.16	8.40

**Table 4:** Description and optimal operation conditions of the enzymes used [31,32].

Commercial Name	Origin	Formulation	pH/Temp. Optimum
Amano Lipase PS	*Burkholderia cepacia*	Immobilized on diatomite	pH 5–9/50 °C
Lipozyme^®^ TL IM	*Thermomyces lanuginosa*	Immobilized on a non-compressible silica gel carrier	pH 6–8/50–75 °C
Lipozyme^®^ Novo 40086	*Rhizomucor miehei*	Immobilized on a resin carrier	pH 7–10/30–50 °C
Lipozyme^®^ RM IM	*Rhizomucor miehei*	Immobilized in beads from microporous anion exchange resins	pH 7–10/30–50 °C
Lipozyme^®^ 435	*Candida antarctica B*	Immobilised on a hydrophobic carrier (acrylic resin)	pH 5–9/30–60 °C

## Data Availability

All data are reported in the paper, any specific query may be addressed to lorena.wilson@pucv.cl.

## Sample Availability

Samples of the compounds available upon request from lorena.wilson@pucv.cl.
